# A Psychosocial Adjustment Measure for Persons With Upper Limb Amputation

**DOI:** 10.33137/cpoj.v5i1.37873

**Published:** 2022-04-05

**Authors:** L.J. Resnik, P. Ni, M.L. Borgia, M.A. Clark

**Affiliations:** 1 Research Department, Providence VA Medical Center, Providence, USA.; 2 Department of Health Services, Policy and Practice, Brown University, Providence, USA.; 3 Biostatistics and Epidemiology Data Analytics Center (BEDAC), Boston University School of Public Health, Boston, USA.; 4 Department of Population and Quantitative Health Sciences, University of Massachusetts Medical School, Massachusetts, USA.

**Keywords:** Upper Limb Amputation, Upper Limb Prosthesis, Psychosocial Adjustment, Factor Analysis, Rasch Analysis, Psychometrics, Prosthesis

## Abstract

**BACKGROUND:**

Measurement of psychosocial adjustment after upper limb amputation (ULA) could be helpful in identifying persons who may benefit from interventions, such as psychotherapy and/or support groups. However, available measures of psychosocial adjustment after limb loss are currently designed for prosthetic users only.

**OBJECTIVE:**

To create a measure of psychosocial adjustment for persons with ULA that could be completed by individuals regardless of whether a prosthesis is use.

**METHODOLOGY:**

We modified items from an existing Trinity Amputation and Prosthesis Experience Survey (TAPES) measure and generated new items pertinent to persons who did not use a prosthesis. Item content was refined through cognitive interviewing and pilot testing. A telephone survey of 727 persons with major ULA (63.6% male, mean age of 54.4) was conducted after pilot-testing. After exploratory and confirmatory factor analyses (EFA and CFA), Rasch analyses were used to evaluate response categories, item fit and differential item functioning (DIF). Item-person maps, score distributions, and person and item reliability were examined. Test-retest reliability was evaluated in a 50-person subsample.

**FINDINGS:**

EFA and CFA indicated a two-factor solution. Rasch analyses resulted in a 7-item Adjustment to Limitation subscale (CFI=0.96, TLI=0.95, RMSEA=0.128) and a 9-item Work and Independence subscale (CFI=0.935, TLI=0.913, RMSEA=0.193). Cronbach alpha and ICC were 0.82 and 0.63 for the Adjustment to Limitation subscale and 0.90 and 0.80 for the Work and Independence subscale, respectively.

**CONCLUSIONS:**

This study developed the Psychosocial Adjustment to Amputation measure, which contains two subscales: 1) Adjustment to Limitation and 2) Work and Independence. The measure has sound structural validity, good person and item reliability, and moderate to good test-retest reliability.

## INTRODUCTION

Individuals experiencing limb loss undergo social and psychological adjustment as well as physical adjustment.^1–4^ Psychosocial adjustment is a process that may involve changes in body image, personal identity, lifestyle and daily functioning.^5^ Psychosocial adjustment can be affected by clinical conditions. A substantial proportion of persons with upper limb amputation experience clinical depression (20-55%),^1,6,7^ post-traumatic stress disorder (23–24%),^1,6^ anxiety (36%),^5^ and long term post-traumatic psychological distress (25%) after amputation.^8^ More than 20% have other co-occurring conditions.^6^ Levels of depression and anxiety are greater and psychosocial adjustment poorer in persons with upper limb amputation (ULA) as compared to those with lower limb amputation,^9^ perhaps due to the visibility of the upper limb and difficulty covering a prosthesis under clothing. Persons with upper limb amputation report an ongoing awareness of difference in appearance,^10^ as well as high levels of body image anxiety and social insecurity that may impact their relationships.^11^ Coping strategies, social support, social discomfort, perceived social stigma, and self-consciousness in public are associated with adjustment to amputation.^5,12^

While several earlier studies utilized qualitative methods to understand psychosocial adjustment after limb loss,^10,11,13–15^ few studies have used quantitative measures of psychosocial adjustment. Studies that did use quantitative measures have been limited to prosthesis users.^5,9,16,17^ Measurement of psychosocial adjustment could be helpful in identifying persons who might benefit from interventions such as psychotherapy and/or support groups. Further, repeated measures of psychosocial adjustment may be needed to understand the experiences and needs of persons with limb loss, given that that adjustment to limb loss is a process that occurs over time.^13^

Unfortunately, to date, there are no measures of psychosocial adjustment that can be used by all persons with ULA. While measures such as the psychosocial adjustment scale of the Trinity Amputation and Prosthesis Experience Survey (TAPES) exist, this measure specifically targets psychosocial adaptation to prosthesis use. The majority of items in this scale refer to use of “an artificial limb”, making it inappropriate for persons with amputation who do not use a prosthesis. Given the prevalence of prosthesis abandonment in ULA (estimated to be between 20-40%),^18,19^ a measure that can be completed by prosthetic users as well as non-users is needed. Thus, the purpose of this study was to create a measure of psychosocial adjustment for persons with ULA that could be completed by individuals regardless of prosthesis use.

## METHODOLOGY

We modified items from the TAPES measure, identified new items, and refined this new item set through cognitive testing and pilot testing. The original TAPES instrument is comprised of three sections (psychosocial adjustment, activity restriction, and satisfaction with prosthesis), each of which contains one or more individually scored subscales, with 15 items related to adjustment.^20,21^ While the TAPES was originally developed for persons with lower limb amputation, the TAPES-ULA was suggested for use in persons with upper limb loss.^22^ The TAPES-ULA, eliminates the item, “*I don’t care if anyone notices I am limping*” due to lack of relevance for persons with upper limb loss. Twelve of the 14 remaining TAPES-ULA items specifically mention the use of an artificial limb. We first made some changes to the original TAPES-ULA items. We changed terminology, replacing the word “*amputation*” with the words “*limb difference*”, to be more inclusive of persons with congenital limb difference. Two items had similar content, “*I don’t mind people asking me about my artificial limb, and I have difficulty in talking about my limb loss in conversation*”, therefore we selected the item that could be completed regardless of prosthesis use. We then added 6 new items that could be completed by both users and non-users of prostheses. The new items were: “*I have adjusted to being an amputee*”, “*I don’t care if somebody looks at my residual arm*”, “*My amputation interferes with the ability to do my work*”, “*Having an amputation makes me more dependent on others than I would like to be*”, “*Having an amputation limits the kind of work that I can do*”, and “*Having an amputation limits the amount of work that I can do*.”

We then administered the item set in cognitive interviews and utilized the feedback to iteratively refine the items and instructions. Cognitive interviewing is an approach commonly used to enhance validity of item content and response processes.^23^ Cognitive interviews were conducted with 11 participants with ULA (9 prosthesis users, 2 non-user). The sample was 63.6% male, with a mean age 54.4 years (**Table 1**). During these interviews, participants were asked to think out loud as they answered the items and to identify any instructions or words that were confusing as well as any questions that were difficult to answer.^24,25^

**Table 1:** Characteristics of the cognitive testing and pilot study samples.

	**Cognitive,** **N=11**	**Pilot,** **N=20**
Age, Mean (SD)	54.4 (9.8)	61.9 (13.5)
**Gender, N (%)**		
Male	7 (63.6)	11 (55.0)
Female	4 (36.4)	9 (45.0)
**Amputation level, N (%)**		
Transradial/wrist disarticulation	5 (45.5)	11 (55.0)
Transhumeral/elbow disarticulation	6 (54.6)	5 (25.0)
Shoulder	0 (0.0)	4 (20.0)
Bilateral upper limb loss	1 (9.1)	4 (20.0)
Prosthesis User, N (%)	9 (81.8)	15 (75.0)
**Primary prosthesis type, N (%)**		
Body powered	6 (66.7)	6 (40.0)
Myoelectric	2 (22.2)	6 (40.0)
Hybrid	0 (0.0)	1 (6.7)
Cosmetic	1 (11.1)	1 (6.7)
Sports/recreation	0 (0.0)	1 (6.7)
**Etiology, N (%)**		
Combat injury	0 (0.0)	2 (10.0)
Accident	2 (27.3)	10 (50.0)
Burn	1 (9.1)	2 (10.0)
Cancer	1 (9.1)	1 (5.0)
Diabetes	0 (0.0)	0 (0.0)
Infection	1 (9.1)	2 (10.0)
Congenital	3 (27.3)	5 (25.0)
Other	2 (18.2)	2 (10.0)
**Race, N (%)**		
White	10 (90.9)	14 (77.8)
Black	1 (9.1)	1 (5.6)
Other	0 (0.0)	3 (16.7)

As a result of feedback obtained in the cognitive interviews, we replaced the word “*residual limb*” with the word “*stump*”, because some respondents in the cognitive interviews told us that this was the terminology that they used most often to refer to their residuum. We also replaced the term “*artificial limb*” with the term “*prosthesis*” because we found that was more commonly used in our sample. We tested the use of the terms “*amputation*”, “*amputee*”, and “*limb difference*.” Because participants with congenital limb difference in our sample did not express any concerns about use of the term “*amputation*” in some items, we retained that language in new items. At the end of cognitive testing, we had a 19-item set. Ten items were specific to prosthesis users, and 9 were pertinent to prosthesis users as well as non-users.

Next, we discussed the measure and instructions with the survey team, who provided feedback based on their prior experiences interviewing individuals with ULA. As a result, we revised the instructions for bilateral participants who were prosthetic users, updating them to refer to the prostheses “*on either side*” instead of “*on your dominant side*.” We also changed the wording of the response options so that the middle of the scale was “*neither disagree nor agree*” (rather than “*neither agree nor disagree*”) to match the directionality of responses within the original psychosocial adjustment TAPES scales. The refined item-set and instructions were then pilot tested in a convenience sample of 20 participants (5 non-users and 15 prosthesis users, 4 persons with bilateral limb loss, 55% male, mean age 61.9) (**Table 1**). No revisions were made to the items or instructions after pilot testing. The final instrument, which was administered in a telephone survey to 727 participants, is shown in **APPENDIX A**.

A subgroup of 50 persons (the reliability sample) completed the telephone survey two times within one week.

### Sample and recruitment

Participants were included if they had amputation at the level of the wrist or above and were able to understand study requirements and hear well enough to comprehend questions administered over the telephone. Participants for all phases of the study (cognitive interviews, pilot testing and field testing) were recruited from several sources: an earlier study conducted in the Department of Veterans Affairs (VA), a list of veterans who had received VA care between January 1, 2016 – June 1, 2019, eblasts sent from the Amputation Coalition of America, and recruitment letters sent from a private prosthetics care company. The study was approved by VA Central institutional review board, and all participants gave oral informed consent as approved by the IRB.

### Data analysis overview

Characteristics of the field study sample were described. To evaluate structural validity of the measure, we performed exploratory and confirmatory analyses using data from the first 351 persons (subsample 1) and final confirmatory analyses using data from the subsequent 376 respondents (subsample 2). We then used data from the entire sample to develop Rasch partial credit models and evaluated item fit statistics, item category curves, and the presence of differential item functioning (DIF). We also evaluated item-person maps, score distributions, and person and item reliability. Finally, we examined test-retest reliability using a convenience subgroup of 50 persons who completed the telephone survey two times within two weeks. These persons were selected based on amputation level and laterality to ensure representation across these key characteristics.

### Factor analyses

The dimensionality of the item pool was evaluated using exploratory factor analysis (EFA) and confirmatory factor analysis (CFA). In the EFA analysis, we utilized factor loadings, eigenvalues, and percentage of variance explained by the first factor to assess unidimensionality for the full 19-item measure. We determined the number of unidimensional factors by identifying the number of eigenvalues > 1 and applying parallel analysis.^26^ We assessed CFA model fit using the comparative fit index (CFI), Tucker–Lewis Index (TLI), root mean square error approximation (RMSEA), and residual correlations. EFA and CFA analyses were conducted with M-Plus software.^27^

We considered CFI and TLI values of 0.90 or higher and RMSEA values <0.08 as acceptable model indices. After examining the model fit for all item combinations, we selected the optimal models that retained the most items with acceptable model fit. We evaluated local independence by inspecting the residual correlations between items; items with residual correlations greater than 0.2 were considered to have local dependence.^28–30^

We then performed CFA using data from subsample 2. Fit criteria used in final confirmatory factor analyses were similar to those used in exploratory analyses. However, rather than using a stringent RMSEA criteria, we focused on examining the residual correlations and eigenvalue ratios rather than on the RMSEA value. We did this because RMSEA is sensitive to the weight matrix, which is used in the Chi-square calculation; a small change in the weight matrix (e.g. different samples) could cause large changes in RMSEA.

### Rasch Partial Credit Modeling and DIF Evaluation

Rasch analyses involve probabilistic modeling of a latent trait, where persons and items are measured on the same interval scale; the Rasch framework allows assessment of psychometric properties for the development and refinement of measures.^31,32^ Rasch partial credit modeling of data from the entire sample was used to evaluate monotonicity, residual variance explained, item fit statistics, item category curves, and the presence of any differential item functioning (DIF). We removed items with “*moderate to large*” DIF (> 0.64) as defined by Zwick.^33^

### Item-person maps and reliability

We used Rasch item-person maps for each factor to evaluate how the range and position of item measure distributions corresponded to the range and position of the person score generated from all items within the factor. We evaluated person reliability (ability to discriminate between persons, or traditional test reliability) as well as item reliability using Rasch models. The test information function was used to determine the ranges of person scores with reliability ≥ 0.9 and ≥ 0.8. Given the smaller number of items completed by non-prosthesis users, we repeated person reliability analyses in the sub-sample that did not utilize a prosthesis. Cronbach’s alpha was used to assess internal consistency of the final factors.

### Transformation and scoring

Rasch summary scores were calculated on a logit scale for the final item set. Person logit scores were then standardized into a T-score matrix, and conversion scoring tables were created (for those with no missing data).

### Test-retest reliability and calculation of minimal detectable change

Fifty participants completed the phone survey twice within 2 weeks (mean 7.8 (SD 3.0), range 3-14 days). These data were used to assess test-retest reliability via the Shrout and Fleiss intraclass correlation coefficient (ICC) type 3,1. We calculated minimal detectable change (MDC) at 90% and 95% confidence levels using the ICC and pooled standard deviation of factor scores (at interviews 1 and 2).

## RESULTS

**Figure 1:**
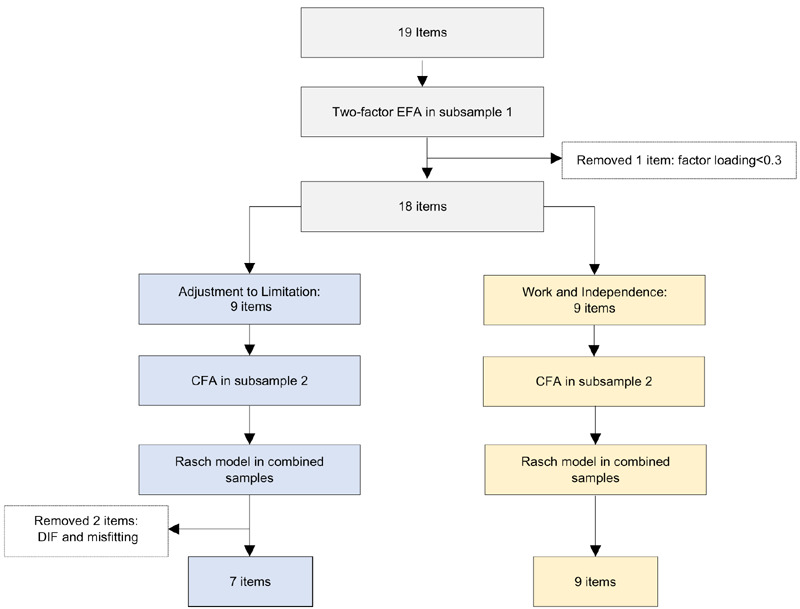
Flow diagram of item reduction and mapping onto factors.

Characteristics of participants in subsample 1, subsample 2, and the test-retest reliability sample are shown in **Table 2**. The item pool utilized in field testing, items not retained, and items in the final Psychosocial Adjustment to Amputation measure are shown in **Table 3**. All decisions for dropping items as a result of EFA and CFA analyses are shown in the **Figure 1** flow chart.

**Table 2:** Characteristics of the field study sample.

	**Subsample 1** **(N=351)**	**Subsample 2** **(N=376)**	**Full Sample** **(N=727)**	**Test-retest Sample** **(N=50)**
**Age-Mean (SD)**	64.0 (12.9)	58.7 (15.8)	61.2 (14.8)	61.1 (14.2)
**Time since amputation-Mean (SD)**	33.1 (18.4)	23.4 (19.7)	28.4 (19.6)	31.7 (19.7)
**Status-N (%)**				
Veteran	334 (95.2)	216 (57.8)	550 (75.9)	47 (94.0)
Military	0 (0.0)	0 (0.0)	0 (0.0)	0 (0.0)
Civilian	17 (4.8)	157 (42.0)	174 (24.0)	3 (6.0)
Unknown	0 (0.0)	1 (0.3)	1 (0.1)	0 (0.0)
**Sex-N (%)**				
Female	10 (2.9)	142 (37.8)	152 (20.9)	2 (4.0)
Male	341 (97.2)	234 (62.2)	575 (79.1)	48 (96.0)
**Race-N (%)**				
White	284 (80.9)	313 (83.2)	597 (82.1)	40 (80.0)
Black	34 (9.7)	32 (8.5)	66 (9.1)	2 (4.0)
Unknown	20 (5.7)	19 (5.1)	39 (5.4)	6 (12.0)
Mixed	13 (3.7)	12 (3.2)	25 (3.4)	2 (4.0)
**Amputation level-N (%)**				
Shoulder	42 (12.0)	33 (8.8)	75 (10.3)	10 (20.0)
Transhumeral	129 (36.8)	97 (25.8)	226 (31.1)	15 (30.0)
Transradial	180 (51.3)	203 (54.0)	383 (52.7)	15 (30.0)
Bilateral	0 (0.0)	43 (11.4)	43 (5.9)	10 (20.0)
**Etiology-N (%)**				
Combat	102 (29.1)	55 (17.2)	157 (23.4)	16 (32.0)
Accident	237 (67.5)	204 (63.7)	441 (65.7)	31 (62.0)
Burn	34 (9.7)	35 (10.9)	69 (10.3)	9 (18.0)
Cancer	15 (4.3)	29 (9.1)	44 (6.6)	3 (6.0)
Diabetes	3 (0.9)	1 (0.3)	4 (0.6)	0 (0.0)
Infection / other health problem	35 (10.0)	71 (22.2)	106 (15.8)	7 (14.0)
Congenital	0 (0.0)	56 (14.9)	56 (7.0)	0 (0.0)
**Current prosthesis user-N (%)**				
Yes	211 (60.1)	261 (69.4)	472 (64.9)	50 (100.0)
No	140 (39.9)	115 (30.6)	255 (35.1)	0 (0.0)
**USERS**				
**Primary prosthesis type-N (%)**				
Body-powered	155 (73.5)	155 (59.4)	310 (65.7)	41 (82.0)
Myoelectric	44 (20.9)	72 (27.6)	116 (24.6)	6 (12.0)
Hybrid	0 (0.0)	4 (1.5)	4 (0.9)	0 (0.0)
Cosmetic	8 (3.8)	20 (7.7)	28 (5.9)	2 (4.0)
Sport	4 (1.9)	6 (2.3)	10 (2.1)	1 (2.0)
Unknown	0 (0.0)	4 (1.5)	5 (0.9)	0 (0.0)
**Hours of daily prosthesis use-N (%)**				
Less than 2 hours	40 (19.1)	45 (17.6)	85 (18.2)	9 (18.0)
2 to less than 4 hours	28 (13.3)	26 (10.1)	54 (11.6)	12 (24.0)
4 to less than 8 hours	38 (18.1)	37 (14.4)	75 (16.1)	5 (10.0)
8 to less than 12 hours	43 (20.5)	62 (24.1)	105 (22.5)	10 (20.0)
12 hours or more	61 (29.1)	87 (33.9)	148 (31.7)	14 (28.0)

**Table 3:** The item pool utilized in field testing, items not retained and items in the final modified Psychosocial Adjustment to Amputation subscales. Note: *Italicized text indicates items limited to prosthesis users.*

**Items used in field testing**	**Item Dropped**	**Final 2-factor satisfaction scales**
		**Factor 1: Adjustment to Limitation**
*I have adjusted to having a prosthesis*		*I have adjusted to having a prosthesis*
*As time goes by, I accept my prosthesis more*		*As time goes by, I accept my prosthesis more*
I feel that I have dealt successfully with this trauma in my life		I feel that I have dealt successfully with this trauma in my life
*Although I have an artificial limb, my life is full*		*Although I have an artificial limb, my life is full*
*I have gotten used to wearing a prosthesis*		*I have gotten used to wearing a prosthesis*
*I don’t care if somebody looks at my prosthesis*	X	
*I find it easy to talk about my prosthesis*		*I find it easy to talk about my prosthesis*
I have difficulty in talking about my limb loss in conversation	X	
I have adjusted to being an amputee		I have adjusted to being an amputee
I don’t care if somebody looks at my stump	X	
		**Factor 2: Work and Independence**
*A prosthesis interferes with the ability to do my work*		*A prosthesis interferes with the ability to do my work*
*Having a prosthesis makes me more dependent on others than I would like to be*		*Having a prosthesis makes me more dependent on others than I would like to be*
*Having a prosthesis limits the kind of work that I can do*		*Having a prosthesis limits the kind of work that I can do*
Being someone with a limb difference means that I can’t do what I want to do		Being someone with a limb difference means that I can’t do what I want to do
*Having a prosthesis limits the amount of work that I can do*		*Having a prosthesis limits the amount of work that I can do*
Having an amputation limits the kind of work that I can do		Having an amputation limits the kind of work that I can do
Having an amputation limits the amount of work that I can do		Having an amputation limits the amount of work that I can do
Having an amputation makes me more dependent on others than I would like to be		Having an amputation makes me more dependent on others than I would like to be
My amputation interferes with the ability to do my work		My amputation interferes with the ability to do my work

### Factor analyses

EFA fit indices for a one-factor solution were not acceptable (CFI=0.83, TLI=0.80, RMSEA=0.15) and eigenvalue ratios for the first and second factor solutions did not suggest a unidimensional scale (9.28/2.84 <4).

Parallel analysis indicated a two components solution. One item (“*I don’t care if somebody looks at my prosthesis*”) had lower factor loadings for both factors (<0.3) and was dropped. This resulted in a two-factor model with 9 items in each factor (Cronbach’s Alpha = 0.90 and 0.84) with acceptable model fit (CFI=0.950, TLI=0.943, RMSEA=0.084).

The RMSEA values for unidimensional model fit for were large for factor 1 (CFI=0.963, TLI=0.950, RMSEA=0.105) and factor 2 (CFI=0.947, TLI=0.930, RMSEA=0.156). Therefore, we further explored unidimensionality by confirming that the ratio of the first and second eigenvalues was >4. We then stratified the sample by prosthesis users (yes vs. no) and repeated the analyses (not shown) to confirm that the factor structure was similar for both prosthesis users and non-users.

CFA fit indices for the unstratified model were (CFI=0.934, TLI=0.925, RMSEA=0.100). RMSEA for each factor were somewhat large (0.138 and 0.193). However, residual correlations were <0.2, eigenvalue ratios were >4, and all eigenvalues except for the first were <1.0. Thus, we concluded that each factor had a unidimensional structure. The factors were labeled based on their content as: **1**) Adjustment to Limitation, and **2**) Work and Independence.

### Calibration and DIF analyses

We used data from the full sample in Rasch partial credit models and examined monotonicity of responses and item calibrations.

In the Adjustment to Limitation factor, monotonicity criteria were not met for the item “*as time goes by, I accept my prosthesis more*.” To address this, we merged the neutral category with the “*agree*” category for factor scoring. After adjustment for this one item, there was no violation of monotonicity. In the Work and Independence factor, the monotonicity criteria were met for all items. In both factors, the neutral response category (“*neither disagree nor agree*”) had a low probability of selection in most items. We explored whether adjusting the model to treat selection of the neutral category as an extra factor fit the data better. We found that the extra factor only affected 6% of respondents, and the adjusted and unadjusted person score correlation was near 1. Conceptually, there was no basis to merge this neutral category with other categories. Therefore, we kept the neutral response category.

In residual factor analysis, unexplained variance in the first contrast was <10% for both factors, and 59.7% and 74.8% of the variance was explained by the measures. Calibrations for both factors are shown in **Table 4**. In the Adjustment to Limitation factor, two items were dropped due to both poor fit and moderate to large DIF by sex: “*I have difficulty talking about limb loss in conversation*” (infit=1.34, outfit=1.67, more difficult for men) and “*I don’t care if somebody looks at my stump*” (infit=1.19, outfit=1.56, more difficult for women). After dropping these items and reanalyzing DIF, we observed that two items had slight to moderate DIF by sex (“*Although I have an artificial limb, my life is full*”) and laterality (“*I find it easy to talk about my prosthesis*”). In the Work and Independence factor, there was slight to moderate DIF by sex (women had more difficulty with “*having an amputation makes me more dependent on others than I would like to be*”). There was also slight to moderate DIF for three items by laterality (those with bilateral amputation had more difficulty).

**Table 4:** Partial credit model of Adjustment to Limitation and Work and Independence Subscales (N=727).

	**Model**		**Infit**		**Outfit**	
	**Measure**	**SE**	**MNSQ**	**ZSTD**	**MNSQ**	**ZSTD**
**Factor 1: Adjustment to Limitation**						
I have gotten used to wearing a prosthesis	41.89	0.32	0.86	-1.5	0.86	-1.4
As time goes by, I accept my prosthesis more	40.97	0.55	1.12	1.2	1.06	0.5
Although I have an artificial limb, my life is full	40.65	0.37	1.16	1.6	1.10	1.0
I have adjusted to having a prosthesis	40.46	0.37	0.90	-1.0	0.75	-2.6
I find it easy to talk about my prosthesis	39.08	0.41	1.19	1.9	1.12	1.2
I feel that I have dealt successfully with this trauma in my life	37.10	0.32	0.91	-1.1	0.91	-1.1
I have adjusted to being an amputee	36.96	0.32	0.92	-1.0	1.32	3.0
**Factor 2: Work and Independence**						
Having an amputation limits the kind of work that I can do	55.36	0.28	1.04	0.7	0.95	-0.6
Having a prosthesis limits the kind of work that I can do	54.02	0.34	1.04	0.5	1.08	0.8
Having an amputation limits the amount of work that I can do	51.79	0.22	0.84	-3.1	0.82	-2.8
Having an amputation makes me more dependent on others than I would like to be	50.67	0.22	0.99	-0.1	1.00	0.0
Having a prosthesis limits the amount of work that I can do	50.17	0.34	0.88	-1.9	0.90	-1.3
Having a prosthesis makes me more dependent on others than I would like to be	50.00	0.34	0.97	-0.4	0.96	-0.5
Being someone with a limb difference means that I can’t do what I want to do	49.22	0.22	1.04	0.7	1.20	3.2
A prosthesis interferes with the ability to do my work	47.88	0.34	1.14	2.2	1.31	4.0
My amputation interferes with the ability to do my work	47.88	0.22	1.09	1.7	1.25	3.9

### Reliability

**Figure 2:**
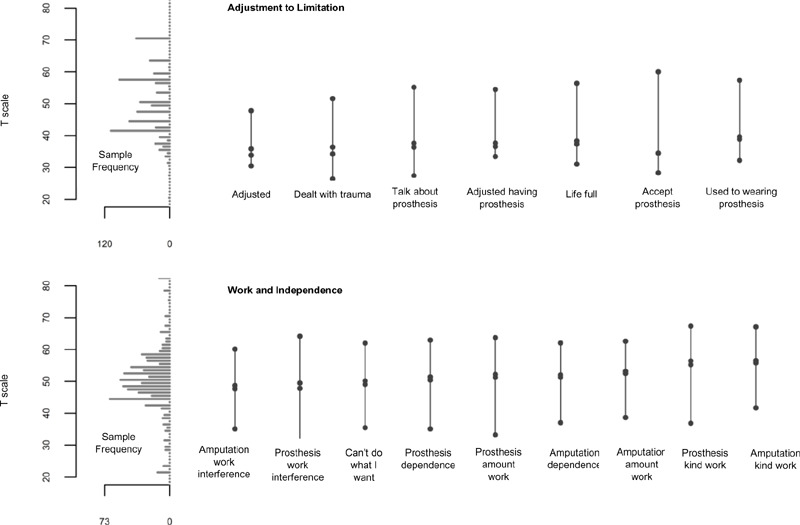
Adjustment to Limitation (top) and Work and Independence (bottom) item-person maps. Note: The histogram of the sample score distribution is shown on the left and level of person ability with 50% probability of selecting each category (vs any higher category) for each item is shown on the right.

Rasch item-person maps for both subscales (**Figure 2**) showed that item difficulties (including the lowest and highest categories) sufficiently covered the range of person ability scores. Rasch reliability correlation coefficients for factor 1 were 0.70 for person reliability and 0.95 for item reliability. In factor 2, the person reliability correlation coefficient was 0.87 and the item reliability correlation coefficient was 0.99.

**Figure 3:**
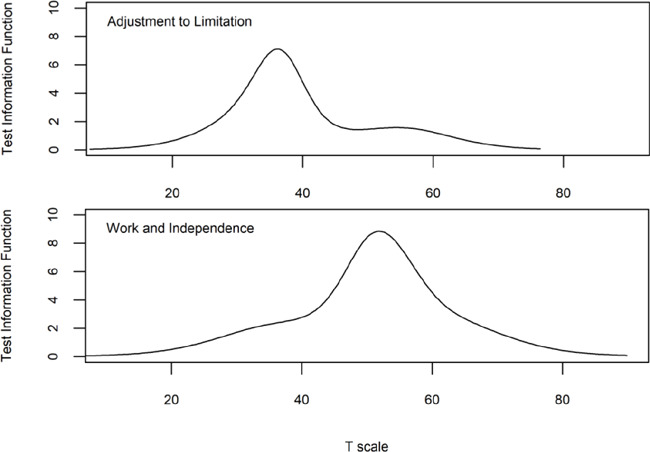
Adjustment to Limitation (top) and Work and Independence (bottom) test information function plots.

Given that non-prosthesis users only completed two items in the Adjustment to Limitation subscale, we examined reliability of this factor for nonusers and found that person reliability was 0.49. However, after removing extreme cases (those with highest or lowest responses for every item), the person reliability was 0.73. In the Adjustment to Limitation subscale, 87% of respondents had scores in the range (22.9 to 61.6) with a score reliability of 0.8 or higher (**Figure 3**). In the Work and Independence subscale, 96% of respondents had scores (29.1 to 70.6) with a reliability at 0.8 or higher (**Figure 3**).

Cronbach alpha was 0.82 and 0.90 for the Adjustment to Limitation and Work and Independence subscales respectively, indicating good internal consistency. ICC (type 3,1) was 0.63 for the Adjustment to Limitation subscale and 0.80 for the Work and Independence subscale indicating moderate and good test-retest reliability, respectively (**Table 5**). MDC 90 and 95 for the Adjustment to Limitation subscale were 17.7 and 14.9, respectively. MDC 90 and 95 for the Work and Independence subscale were 13.0 and 10.9, respectively.

**Table 5:** Psychosocial Adjustment to Amputation Subscale ICCs and MDCs.

	**N**	**Interview 1: Mn (SD)**	**Interview 2: Mn (SD)**	ICC (95% CI)*	**MDC 90**	**MDC 95**
**Adjustment to Limitation**	50	51.5 (10.7)	52.6 (10.2)	0.63 (0.42, 0.77)	14.9	17.7
**Work and Independence**	50	50.6 (10.4)	51.1 (10.4)	0.80 (0.67, 0.88)	10.9	13.0

* CI: confidence interval, ICC: intraclass correlation coefficient, MDC: minimal detectable change. Note: We interpreted reliability with ICC <0.50 considered poor 0.50-0.75 moderate, 0.75-.90 good and >0.90 excellent. ^34^

## DISCUSSION

Our objective was to create a psychosocial adjustment scale for persons with ULA that could be used in research and clinical practice for upper prosthesis users and non-users alike, overcoming a limitation of the TAPES-ULA psychosocial adjustment subscale which is targeted to prosthesis users only. The strengths of this study include the large sample size and a robust sample of women with ULA. The measure that we developed could be used as a screening tool in clinical practice to identify persons with poor psychosocial adjustment who might benefit from referral to behavioral health care providers. A copy of the final measure is provided in **APPENDIX B**.

Given our sample size and characteristics, we were able to evaluate the extent of differential item functioning across key population categories. We field tested a 19-item set in a sample that included 472 prosthetic users and 255 non-users. The new Psychosocial Adjustment to Amputation measure we developed contains two subscales: a 7-item Adjustment to Limitation scale and a 9-item Work and Independence subscale. The new subscales had sound structural validity, good person and item reliability, and moderate to good test-retest reliability. No retained items had moderate to large DIF by sex, age, prosthesis use, or laterality.

Our work has application for future studies of psychosocial adjustment to ULA. Prior studies in this area were limited to persons who use a prosthesis.^9,16^ We provided estimates of minimal detectable change (MDC) that can be used when interpreting change in scores at an individual level in longitudinal studies of psychosocial adjustment.

While we believe that our tool is useful in its present state, further research is needed to enhance reliability of the Adjustment to Limitation subscale. This scale contains only two items that can be completed by persons who do not use a prosthesis and as such has lower reliability for non-users. This finding suggests that this subscale should be used cautiously for persons who do not use a prosthesis and that it may not be appropriate for use at the person level for determining change in Adjustment to Limitation in persons who do not use a prosthesis. In contrast, the Work and Independence subscale contains 5 items that are pertinent to persons who do not use a prosthesis and had stronger reliability. We recommend that additional items be developed and added to the Adjustment to Limitation subscale to enhance its reliability.

### Limitations

Our study has several limitations. First, our study activities were conducted in English language only and with persons from the United States. Our findings need to be replicated with translated versions and with English speaking persons from other countries.

Second, males in our sample were predominantly veterans, while the majority of women were non-veterans. Despite this difference, we found only minor differential item functioning by sex in the Work and Independence Subscale. Although we evaluated differential item functioning at the item level, this study did not compare overall scale scores by sex. These types of comparisons will need to be conducted in future studies. Our findings may be limited to older persons, given that the mean age of our sample was 61, suggesting findings may not be representative of younger persons. Additionally, the sample was predominately white and non-Hispanic, limiting the generalizability to persons from less diverse backgrounds. Further research is needed to assess how findings may vary by race and ethnicity.

Another limitation of the study is that all data were collected by interviewer administration by telephone. This mode of administration was selected to facilitate response rates and reduce respondent burden, given that the psychosocial item set was administered as part of a larger study of participant-reported measures, with interviews lasting about 45 minutes. We acknowledge that respondents did not have copies of the measures or response categories. We made special efforts to customize the instructions to make telephone administration clear and study interviewers were carefully trained, professional survey staff members. Further research is needed to examine the revised measure when administered by paper and pencil or electronically, and to compare results by mode of administration.

We provided estimates of test-retest reliability and estimates of MDC values. Our test-retest subsample was only 4% female while the overall sample was 20% female. Therefore, these data should be considered preliminary and limited to men with ULA.

## CONCLUSION

We developed a new tool to measure psychosocial adjustment in persons with ULA and examined its measurement properties. The result was a 7-item Adjustment to Limitation subscale and a 9-item Work and Independence subscale with sound structural validity, good reliability, and evidence supporting test-retest reliability. Both scales can be used for persons with ULA to assess the important construct of psychosocial adjustment and identify persons who might benefit from behavioral health referral regardless of whether they use a prosthesis. However, the Adjustment to Limitation subscale has marginal reliability in the nonuser subgroup.

## DECLARATION OF CONFLICTING INTERESTS

All authors declare that they have no conflict of interests.

## AUTHOR CONTRIBUTION

**Linda J. Resnik**:

Conceptualization, data interpretation, manuscript, writing, project administration, funding acquisition.

**Pengsheng Ni**:

Statistical methodology, data analysis, visualization and interpretation.

**Matthew L. Borgia**:

Statistical methodology, data analysis, visualization and interpretation, writing and editing.

**Melissa A. Clark**:

Study design, survey design, data interpretation, manuscript review and revision.

## SOURCES OF SUPPORT

Department of Veterans Affairs Rehabilitation Research and Development Service A2936-R and A9264-S. Sponsors had no role in study design, collection, analysis, and interpretation of data.

## ETHICAL APPROVAL

The study was approved by VA Central institutional review board, and all participants gave oral informed consent as approved by the IRB.

## APPENDIX A

**Measure Used in Field Testing**

Thinking about your upper limb amputation, how much do you disagree or agree with each of the following statements?

**Table:**

	Strongly Disagree	Disagree	Neither Disagree nor Agree	Agree	Strongly Agree	DON’T KNOW/NOT SURE [DO NOT READ]	REFUSED [DO NOT READ]
a. I have adjusted to being an amputee [Would you say Strongly Disagree, Disagree, Neither Disagree nor Agree, Agree, or Strongly Agree?]	1	2	3	4	5	98	99
b. I feel that I have dealt successfully with this trauma in my life	1	2	3	4	5	98	99
c. I don’t care if somebody looks at my stump	1	2	3	4	5	98	99
d. I have difficulty in talking about my limb loss in conversation	1	2	3	4	5	98	99
e. My amputation interferes with the ability to do my work	1	2	3	4	5	98	99
f. Having an amputation limits the **kind** of work that I can do	1	2	3	4	5	98	99
g. Having an amputation limits the **amount** of work that I can do	1	2	3	4	5	98	99
h. Having an amputation makes me more dependent on others than I would like to be	1	2	3	4	5	98	99
i. Being someone with a limb difference means that I can’t do what I want to do	1	2	3	4	5	98	99

Thinking about your prosthesis, how much do you disagree or agree with each of the following statements?

**Table:**

	Strongly Disagree	Disagree	Neither Disagree nor Agree	Agree	Strongly Agree	KNOW/NOT SURE [DO NOT READ]	REFUSED [DO NOT READ]
a. I don’t care if somebody looks at my prosthesis [Would you say Strongly Disagree, Disagree, Neither Disagree nor Agree, Agree, or Strongly Agree?]	1	2	3	4	5	98	99
b. I have adjusted to having a prosthesis	1	2	3	4	5	98	99
c. As time goes by, I accept my prosthesis more	1	2	3	4	5	98	99
d. Although I have an artificial limb, my life is full	1	2	3	4	5	98	99
e. I have gotten used to wearing a prosthesis	1	2	3	4	5	98	99
f. I find it easy to talk about my prosthesis	1	2	3	4	5	98	99
g. A prosthesis interferes with the ability to do my work	1	2	3	4	5	98	99
h. Having a prosthesis limits the **kind** of work that I can do	1	2	3	4	5	98	99
i. Having a prosthesis limits the **amount** of work that I can do	1	2	3	4	5	98	99
j. Having a prosthesis makes me more dependent on others than I would like to be	1	2	3	4	5	98	99

## APPENDIX B

**Psychosocial Adjustment to Amputation Measure**

1.Thinking about your upper limb amputation, please indicate how much you disagree or agree with each of the following statements.

**Table:**

	Strongly Disagree	Disagree	Neither Disagree nor Agree	Agree	Strongly Agree
**Adjustment to Limitation**					
I have adjusted to being an amputee	1	2	3	4	5
I feel that I have dealt successfully with this trauma in my life	1	2	3	4	5
**Work and Independence**					
My amputation interferes with the ability to do my work	5	4	3	2	1
Having an amputation limits the **kind** of work that I can do	5	4	3	2	1
Having an amputation limits the **amount** of work that I can do	5	4	3	2	1
Having an amputation makes me more dependent on others than I would like to be	5	4	3	2	1
Being someone with a limb difference means that I can’t do what I want to do	5	4	3	2	1

2. Thinking about your prosthesis, please indicate how much you disagree or agree with each of the following statements.

**Table:**

	Strongly Disagree	Disagree	Neither Disagree nor Agree	Agree	Strongly Agree
**Adjustment to Limitation**					
I have adjusted to having a prosthesis	1	2	3	4	5
As time goes by, I accept my prosthesis more	1	2	3	3	4
Although I have an artificial limb, my life is full	1	2	3	4	5
I have gotten used to wearing a prosthesis	1	2	3	4	5
I find it easy to talk about my prosthesis	1	2	3	4	5
**Work and Independence**					
A prosthesis interferes with the ability to do my work	5	4	3	2	1
Having a prosthesis limits the **kind** of work that I can do	5	4	3	2	1
Having a prosthesis limits the **amount** of work that I can do	5	4	3	2	1
Having a prosthesis makes me more dependent on others than I would like to be	5	4	3	2	1
